# Capsulotomy morphology in cataract surgery: comparison of five femtosecond lasers

**DOI:** 10.1097/j.jcrs.0000000000001758

**Published:** 2025-08-14

**Authors:** Isabel A. Joia, Jonas M. Dürmüller, Willi Halfter, Carsten H. Meyer, Paul Bernhard Henrich

**Affiliations:** From the University of Basel, Basel, Switzerland (Joia); Università della Svizzera Italiana, Lugano, Switzerland (Dürmüller, Henrich); Department of Ophthalmology, University of Basel, Basel, Switzerland (Halfter); Augenzentrum Grischun, Davos, Switzerland (Meyer); Department of Ophthalmology, University of Marburg, Germany (Meyer); Oftacentro, Lugano, Switzerland (Henrich).

## Abstract

**Purpose:**

To compare the capsulotomy rim morphology of anterior lens capsules obtained by 5 femtosecond laser–assisted cataract surgery (FLACS) platforms: Catalys, FEMTO LDV Z8, LensAR, LenSx, and Victus.

**Setting:**

University of Basel, Basel, Switzerland.

**Design:**

Experimental study.

**Methods:**

Anterior lens capsules from 6 interventions per laser platform were collected (N = 30). 4 of each were assessed under light microscopy (LM; n = 20), and 2 were assessed under scanning electron microscopy (n = 10). A qualitative evaluation of all samples was conducted, as well as a semiquantitative analysis of the extent of irregularities present in LM samples. The Dunn multiple comparisons test was conducted to determine which lasers produced significantly smoother capsulotomies.

**Results:**

Irregularities along the lens capsule rim were observed in all samples. Distinct irregularities were observed for Catalys (unconnected laser pulses bordering the capsulotomy), FEMTO LDV Z8 (numerous additional rows of laser pulses), and LensAR (at least one section with multiple rows of laser pulses). Victus demonstrated a significantly higher degree of smoothness of capsulotomy (median = 306 degrees) compared with Catalys (median = 171 degrees) (*P* = .02). The medians for other laser platforms fell between these values.

**Conclusions:**

Different laser platforms produce morphologically and quantitatively distinct irregularities, which may influence capsulotomy strength differently. FEMTO LDV Z8, a low-energy laser, did not produce a smoother capsulotomy than the high-energy lasers. Our results suggest that, with current technologies, the precision of the laser is more important than the pulse energy in determining the regularity of the capsulotomy surface.

## Abstract 2

The evaluation of 30 capsulotomies from 5 FLACS platforms showed that different lasers cause distinct morphologic irregularities and that the regularity of the capsulotomy depends mainly on laser precision.

Cataract surgery is one of the most frequently performed surgeries, with an estimated over 7 million yearly interventions in the United States and the European Union combined.^1,2^ The introduction of femtosecond laser–assisted cataract surgery (FLACS) over a decade ago presents an important advancement in surgical techniques for cataract treatment.^3^ This procedure is characterized by the use of a laser to perform capsulotomy compared with a fine needle or forceps during conventional phacoemulsification cataract surgery (CPCS) (Figure 1, A – D).^4^ This difference in technique results in distinct effects on the shape and morphology of the capsulotomy. On a macroscopic level, FLACS capsulotomies are positioned more accurately and are rounder in shape compared with CPCS capsulotomies, leading to a better centration of the intraocular lens in the capsular bag (compare Figure 1, A and C).^5–8^ However, on a microscopic level, FLACS capsulotomy rims are irregular, characterized by tags, aberrant laser pulses, indentations, and notches.^9,10^ By contrast, CPCS capsulotomy rims are smooth and homogeneous (compare Figure 1, B and D).^11^

**Figure 1.**
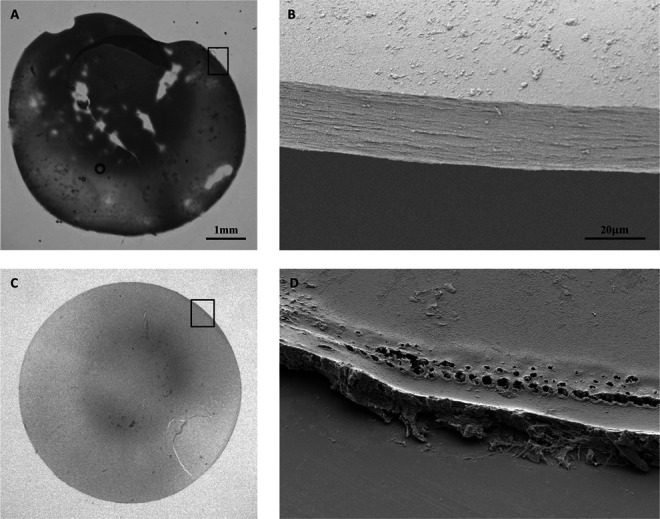
*A*: Extracted CPCS lens capsule under LM. Clear areas on the lens capsule are a result of incomplete staining. Black squares indicate the lens capsule rim. *B*: SEM image of CPCS lens capsule rim at ×1000 magnification. *C*: Extracted FLACS lens capsule under LM. *D*: SEM image of FLACS lens capsule rim at ×1000 magnification. While macroscopically, FLACS capsulotomies are more centered and circular, irregularities along the capsulotomy circumference can be seen at a microscopic level. CPCS = conventional phacoemulsification cataract surgery; LM = light microscopy; SEM = scanning electron microscopy

The microscopic irregularities seen in FLACS capsulotomies may influence surgical outcomes. For instance, discontinuous laser pulses could result in an incomplete separation of the created flap from the anterior lens capsule.^12^ In addition, the irregularities lead to an uneven stress distribution along the capsulotomy's circumference with focal points of high mechanical stress, which weaken the anterior capsule rim.^13–15^ Both adherence of the capsular flap and reduced rhexis stability may predispose the anterior lens capsule to tearing during manipulation.^16^ Anterior capsular tears are, in turn, associated with an elevated risk for severe complications, including zonular damage, posterior extension of the capsular rupture, and vitreous loss.^17,18^ To minimize these risks, FLACS systems should ideally produce capsulotomy edges that are as smooth and as regular as possible.

Various factors have been hypothesized to contribute to the formation of microscopic irregularities in FLACS, among which pulse energy has been postulated to be a significant determinant. Higher pulse energies are hypothesized to cause greater tissue disruption and have been associated with a more irregular capsulotomy in previous studies, whereas lower pulse energies have been associated with smoother capsulotomy edges.^9,19,20^ This distinction has given rise to the concept of high-energy and low-energy femtosecond lasers, with low-energy lasers operating at pulse energies in the nanojoule range being marketed as leading to less tissue damage and fewer anterior capsule complications, although this remains to be conclusively demonstrated.^20,21^

Previous research has primarily focused on comparing the capsulotomy morphology between CPCS and FLACS and on assessing the impact of varying pulse energies on capsulotomy morphology in theoretical and in vitro studies. However, little is known about how different FLACS platforms affect capsulotomy morphology in patient-derived samples. The goal of this study was to compare the microanatomical surface structure of patient-derived capsulotomy samples among 5 different FLACS platforms. In addition, this study aimed to test the hypothesis that low-energy FLACS devices produce a smoother capsulotomy edge compared with high-energy FLACS devices in patient-derived samples.

## METHODS

Ethics approval was obtained from the Ethics Committee of Northwestern and Central Switzerland. The study was conducted in adherence with the tenets of the Declaration of Helsinki at the Eye Hospital of the University of Basel. Informed consent was obtained from all patients. Patients undergoing FLACS were included in our study. Patients younger than 18 years old, with traumatic cataract injuries, hypermature cataracts, or pseudoexfoliation syndrome were excluded.

FLACS was performed in 5 different centers by 5 different surgeons, each center using a specific laser system (Catalys, Johnson and Johnson, FS, OP-Zentrum Bahnhof Basel, Basel, Switzerland; FEMTO LDV Z8, Ziemer, CE, Medizentrum Eckert, Herrenberg, Germany; LensAR, LensAR, Topcon, DB, Breyer, Kaymak & Klabe Augenchirurgie, Dusseldorf, Germany; LenSx, Alcon, HM, Mainblick Augenzentrum, Aschaffenburg, Germany; and Victus, Bausch and Lomb, HB, Augenarztzentrum Zürich, Zurich, Switzerland). Customizable capsulotomy parameters across laser systems were set according to the surgeon's preference (Catalys: capsulotomy diameter [CD] = 5 mm, resection height [RH] = 0.4 mm, horizonal spot spacing = 5 μm, vertical spot spacing = 15 μm, laser time = 1.7 seconds; FEMTO LDV Z8: CD = 5.5 mm, RH = 1.2 mm, capsulotomy power = 100%; LensAR: CD = 5 mm, RH = 0.4 mm, pulse energy = 5 μJ; LenSx: CD = 4.9 mm, pulse energy = 5.8 μJ, capsule anterior depth = 3.76 mm, capsule posterior depth = 4.04 mm, spot separation = 4 μm, layer separation = 3 μm; and Victus: CD = 5 mm, RH = 1 mm, pulse energy = 5.5 μJ). Capsulotomies were performed before phacoemulsification. FEMTO LDV Z8 was the only low-energy laser tested. Per manufacturer specification, FEMTO LDV Z8 uses pulse energies in the nanojoule range, whereas the other laser systems apply pulse energies in the microjoule range.^21^

For each laser system, 6 extracted anterior lens capsules, which are normally discarded after FLACS, were collected between August 2017 and June 2023 (N = 30). The lens capsules were suspended in a vial containing phosphate-buffered saline and stored at 6°C. At no point during sample preparation was glutaraldehyde used for fixation. Our previous attempts to fixate anterior capsule flaps with glutaraldehyde demonstrated that it modified the architecture of the capsulotomy edge. In SEM analysis, the capsule margin of samples fixated with glutaraldehyde appeared smoothened, and the notches and grooves normally observed along the whole capsulotomy edge in FLACS samples were no longer discernible. Storage in phosphate-buffered saline maintained the physiological conditions and did not introduce artifacts at the capsule edge.^22^

### Preparation

All lens capsules were mounted with the epithelial side facing upward onto cover slips coated with poly-L-lysine and centrifuged for 5 minutes at 1000 rpm. The lens capsules were randomly assigned to 2 groups: 4 samples per laser system were examined under light microscopy (LM; n = 20) and 2 under scanning electron microscopy (SEM; n = 10).

For LM analysis (Zeiss), samples were prepared with a drop of Vectashield Antifade Mounting Medium and covered with a cover glass for evaluation.

For SEM analysis, the lens capsules were dehydrated through an ascending ethanol series and subsequently dried using the Autosamdri 815 Critical Point Dryer (Tousimis Research Corp.). A 20 nm gold coating was then applied using Leica EM Ace 600 (Leica Microsystems GmbH). The samples were examined under SEM (Philips XL30 ESEM, Philips Medical Systems Nederland B.V.), with the entire capsule rim viewed at 2500-fold and 1000-fold magnifications.

### Analysis

A qualitative as well as a semiquantitative analysis of the capsulotomies was conducted. For qualitative analysis, the appearance of the irregularities observed along the entire lens capsule circumference was examined and described for all samples by 1 observer (I.J.).

**Figure 2.**
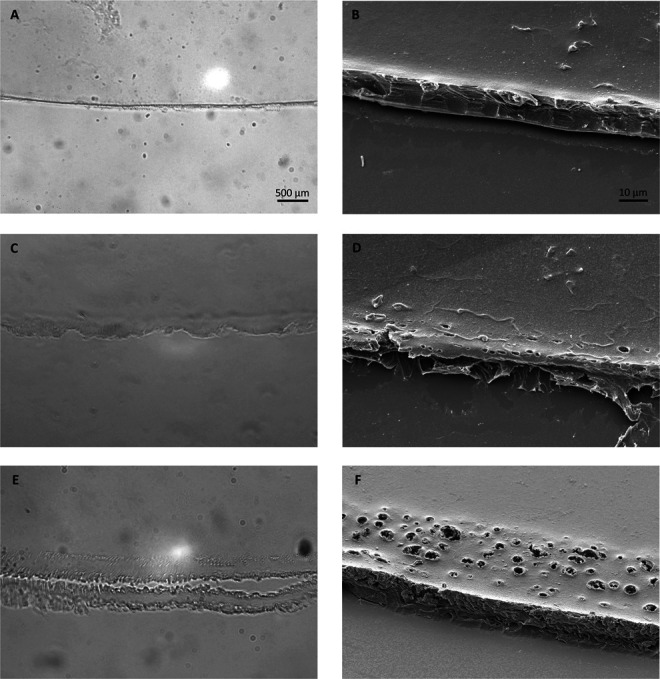
Representative images for the grades of regularity used to label the capsule edge under LM (×100 magnification) and an equivalent SEM image (×1000 magnification). *A*: Smooth, LM. *B*: Smooth, SEM. *C*: Intermediate, LM. *D*: Intermediate, SEM. *E*: Irregular, LM. *F*: Irregular, SEM. LM = light microscopy; SEM = scanning electron microscopy

For semiquantitative analysis, 3 distinct grades of regularity were assigned to the circumference of the lens capsules observed under LM (n = 20) at 100-fold magnification (Figure 2). Lens capsule margins were labelled smooth, intermediate, or irregular. Smooth was defined as a continuous, regular edge (Figure 2, A and B). Intermediate was defined as edges displaying slight irregularities with a single or multiple rows of notches and perforations located within a visually estimated distance of 20 μm from the edge (Figure 2, C and D). Irregular was defined by coarse irregularities along the edge with multiple rows of deviating laser pulses at a visually estimated distance greater than 20 μm from the edge (Figure 2, E and F). The label for every section of the lens capsule was manually mapped onto a circle and converted into angular measurements expressed in degrees. To minimize bias, 3 experts masked to the platform (W.H., I.J., J.D.) evaluated each sample.

Descriptive statistics were conducted in Microsoft Excel (v. 2409, Microsoft Corp.) and comparative statistics in R (v. 4.4.0, R Core Team). Mean imputation was applied to replace missing data. The mean values of the 3 independent evaluations for every sample were used for statistical analysis.

Interrater reliability as intraclass correlation coefficient estimates and their 95% confidence intervals were calculated based on a single rater, absolute-agreement, 2-way random-effects model.^23^ As the sample size per laser system was small, nonparametric testing was used. The median and interquartile range for each label of every laser system was calculated. A Kruskal-Wallis test was conducted to determine whether the degrees labelled smooth differed among the laser systems. Subsequently, a post hoc Dunn multiple comparisons test was conducted to determine which laser systems had significantly different proportions of smooth capsulotomy edges. The experiment-wise error rate was adjusted using Bonferroni correction. The significance level was set to *P* < .05.

## RESULTS

### Qualitative Analysis

**Figure 3.**
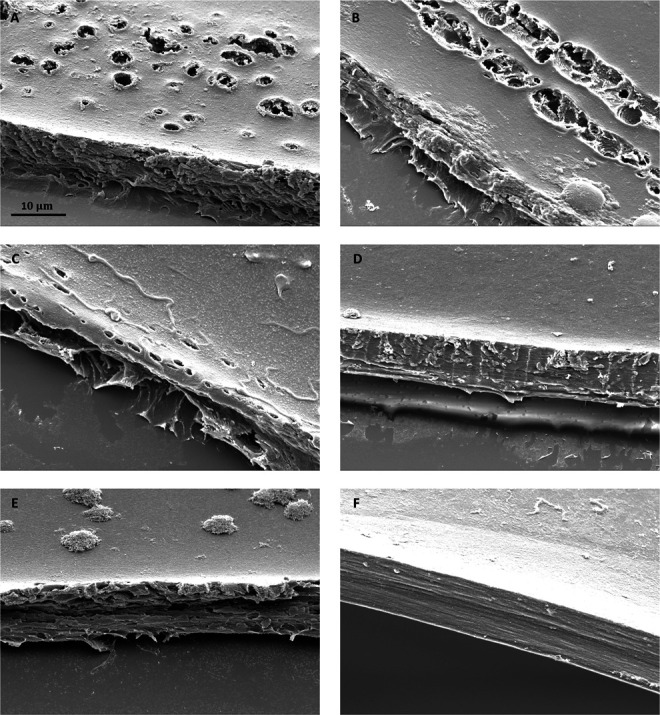
SEM images of capsule edges at 1000-fold magnification. *A*: A fringe of unconnected aberrant laser pulses along the circumference of the capsulotomy, which is only seen in Catalys samples. *B*: Two rows of additional connected laser pulses running parallel to the capsulotomy circumference in an FEMTO LDV Z8 sample. This irregularity is typical of this laser. *C*: Singular misdirected laser pulses in the proximity of the capsule edge in a LensAR sample. *D*: Slight indentations along the cut surface of a LenSx sample caused by laser pulses. *E*: Cut surface with notches in a Victus sample. *F*: A crack in the lens capsule of a FEMTO LDV Z8 sample that occurred during dehydration for SEM analysis and must not be mistaken for a smooth and homogenous capsulotomy rim. SEM = scanning electron microscopy

All 30 lens capsules were complete and circular, except for 1 Victus sample, where 22% (0.75% of total data) of the circumference was damaged after capsulotomy and could not be examined. The appearance of 20 lens capsules was evaluated under LM and 10 under SEM. Irregularities seen under LM were comparable with the ones observed under SEM (compare Figure 2, A, C, E, with Figure 2, B, D, F, respectively). Examined samples of all laser systems exhibited singular aberrant laser pulses, rows of additional connected laser pulses, and indentations and notches along the cut surface (Figure 3, A-E). In addition to these irregularities common to all laser systems, Catalys, FEMTO LDV Z8, and LensAR samples exhibited distinct morphological characteristics.

All Catalys samples showed a unique morphological characteristic consisting of unconnected aberrant laser pulses, forming a fringe up to 80 μm wide along the capsule's perimeter for approximately half of the capsule's circumference, which gradually transitioned into a smooth edge without irregularities (Figure 3, A). This distinctive feature was the main irregularity observed in Catalys samples.

The characteristic irregularity in FEMTO LDV Z8 samples was multiple additional rows of connected laser pulses of varying lengths, some extending up to 800 μm, that cut into the lens capsule at steep, sometimes perpendicular angles, running parallel to the circumference of the capsulotomy (Figure 3, B). By contrast, lens capsules of other lasers exhibited fewer and shorter additional rows of perforations, rendering the FEMTO LDV Z8 samples distinguishable from those of other laser devices.

All LensAR samples consistently exhibited at least one section featuring either multiple rows of laser pulses up to 70 μm wide and running parallel to the capsulotomy circumference or poorly detached lens capsule material adhering to the capsulotomy margin.

When examining SEM samples, it is important to note that the lens capsule may crack during dehydration. Along the crack, the cut surface appears homogeneous and smooth, similar to a manually performed capsulotomy (Figures 1, B and 3, F). It is crucial not to mistake these preparation artifacts for a smooth capsulotomy surface.

### Semiquantitative Analysis

Twenty lens capsules were examined under LM. 22% of the circumference of a Victus sample was missing (1.1% of LM data). The proportion of the circumference labelled smooth, intermediate, or irregular for the 20 samples was measured in degrees by 3 independent observers. The interrater reliability was 0.8 (95% CI 0.71-0.87).

**Figure 4.**
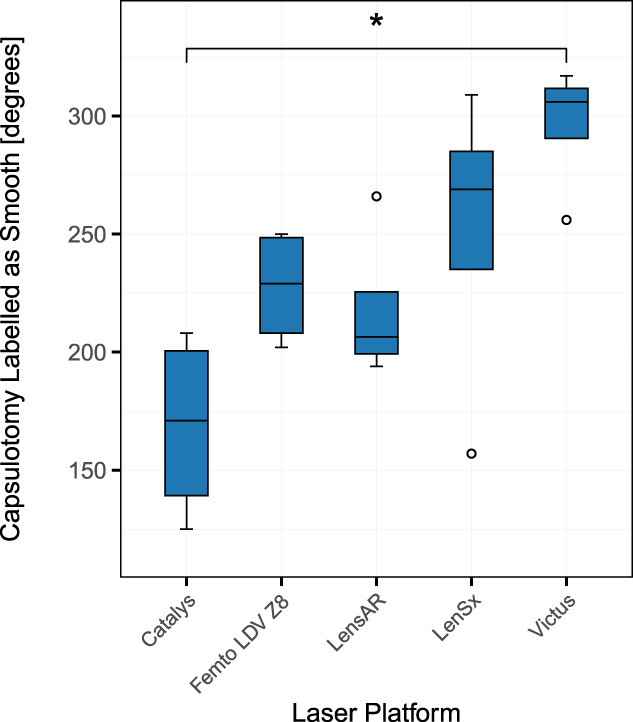
Results of the semiquantitative analysis. The box plot of circumference labelled smooth for every laser system (in degrees) is depicted. The asterisk indicates a significant difference (*P* < .05) in the Dunn test for multiple comparisons.

Table 1 depicts the results of the semiquantitative analysis. The median and interquartile range in degrees for each classification of every laser system was calculated (Figure 4). The Kruskal-Wallis test revealed that there was a statistically significant difference in the degree of edges labelled smooth between at least 2 groups (*P* = .03). Pairwise comparisons using the Dunn test found that the degree of smooth edges was significantly different between Victus and Catalys (*P* = .02). The other differences were not statistically significant (*P* > .36).

**Table 1.** Results of the semiquantitative analysis

Grade of regularity	Catalys (degrees)	FEMTO LDV Z8 (degrees)	LensAR (degrees)	LenSx (degrees)	Victus (degrees)
Irregular	94 (71, 120)	34 (18, 47)	40 (38,47)	5 (0, 35)	0 (0, 0)
Intermediate	96 (76, 114)	93 (81, 113)	103 (88, 111)	82 (67, 94)	53 (49, 69)
Smooth	171 (140, 200)	230 (208, 249)	207 (200, 226)	270 (235, 285)	306 (291, 310)

## DISCUSSION

The results of the qualitative analysis demonstrate that while some irregularities are common to all laser platforms, certain laser systems create typical morphologic characteristics that render them distinguishable from the others. These include a fringe of unconnected laser pulses for Catalys, numerous additional rows of laser pulses for FEMTO LDV Z8, and at least 1 particularly irregular section with multiple rows of laser pulses in all examined LensAR samples. Based on their appearance, the observed irregularities are most likely unrelated to the amount of pulse energy, which mainly influences the size of the perforation produced by the singular laser pulses.^20^ The additional rows of pulses and the aberrant singular pulses are misdirected laser pulses deviating from the capsulotomy line. This suggests that the observed irregularities are caused by a lack of precision of the laser, defined as the discrepancy between the observed laser pulses and a hypothetically perfectly circular capsulotomy, or by factors hindering the precise application of laser pulses.

In our semiquantitative analysis, Catalys was found to produce a significantly lower proportion of regular capsular margins as compared with Victus. To put this result into perspective, the impact of the observed irregularities on the anterior capsule strength and on the likelihood of capsular tears must be considered.^13–15^ Some patterns of morphological irregularities potentially have a stronger influence on anterior capsule strength than others. The typical irregularity seen in Catalys samples was many unconnected laser pulses bordering the capsulotomy circumference. From a mechanical perspective, we hypothesize that unconnected laser pulses generate lower focal stress concentrations along the capsulotomy circumference during manipulation than rows of connected laser pulses. Lasers that produce many additional rows of laser pulses, such as FEMTO LDV Z8, may therefore have a more detrimental effect on rhexis stability than lasers that produce unconnected aberrant laser pulses, such as Catalys.

Based on both the qualitative and semiquantitative analyses, Victus showed the smoothest capsulotomy margins, whereas Catalys and FEMTO LDV Z8 displayed the highest degree of rhexis margin irregularities: Catalys produced the least amount of smooth capsulotomy margins, and FEMTO LDV Z8 samples exhibited morphologic characteristics that could compromise the anterior capsule strength. LenSx's and LensAR's performances fell in between.

To date, relatively few studies have compared the morphology of the anterior lens capsule across different FLACS platforms, and those available are often limited by small sample sizes. Our findings are consistent with those reported by Tognetto et al., who observed that Catalys generated more irregular capsulotomy margins compared with LenSx.^5^ In other comparative studies, no statistically significant difference between different FLACS platforms was found.^24–26^ This, however, might be attributed to a lack of statistical power. Previous literature has not thoroughly investigated the specific morphological patterns characteristic of different femtosecond laser systems. Nevertheless, in our study, FEMTO LDV Z8 samples exhibited numerous additional rows of laser pulses, a finding consistent with observations reported by Mayer et al.^27^ Overall, further comparative studies with larger sample sizes are necessary to validate our findings.

Previous theoretical and in vitro studies support the hypothesis that low-energy lasers produce a smoother capsulotomy edge compared with high-energy lasers. FLACS lasers operating at lower energies have been shown to achieve a more regular capsulotomy edge and a higher anterior capsule strength.^6,9,19,20^ Furthermore, in a study by Lin et al., FEMTO LDV Z8, a low-energy laser, allowed for a better detachment of the anterior capsule flap after capsulotomy in patients as compared with LenSx, a high-energy laser.^21^ However, in our study, FEMTO LDV Z8 showed lower smoothness of capsulotomy compared with other lasers (even if not statistically significant). This contradictory evidence may have various possible explanations.

One possible explanation is that the previously described superior detachment in FEMTO LDV Z8 capsulotomy flaps may have resulted from a more intense cutting method of the laser. Another possible explanation is that in vitro studies and studies on the mechanical physics behind femtosecond lasers fail to take into account external factors that influence the precision of the laser in clinical practice, such as eye fixation movements during surgery or interactions between the laser interface and the patient's cornea.^27^ For example, applanating patient interface technologies that flatten the cornea and subsequently change the refractive angles of the cornea and distort the laser beam may be partially responsible for the imprecise application of laser pulses.^28,29^ Loss of suction and detachment of the patient interface may also negatively affect capsulotomy.^30^ Such patient-related factors can only be accounted for through studies on patient-derived samples, such as the one presented in this article. Another important aspect to be considered is that every FLACS platform uses different software, imaging technology, laser devices, and different customizable laser parameters, all of which may influence laser accuracy. Although theoretically, low-energy FLACS produce a smoother capsulotomy, the precision of the laser and the interactions of the platforms with the patient have to be taken into account to evaluate real-life capsulotomy outcomes.

A key limitation of this study was the small number of samples analyzed per laser system. Another source of potential bias was that the laser parameters were set according to the surgeons' preferences, with only one surgeon operating each laser system. Customizable laser parameters are not the same for every laser, and setting the parameters of different FLACS platforms to equal values to limit parameter bias is difficult to implement. One possibility would have been to set the parameters to their standard settings, which reflect the ideal capsulotomy parameters according to manufacturers, when comparing different FLACS platforms. Another important consideration is that our study solely analyzed the anterior capsule flap that was extracted after FLACS. We assumed that irregularities along the capsulotomy margin were symmetrical, implying that similar irregularities would have been present in the remaining anterior capsule in the patient's eye. However, this assumption may not necessarily be accurate.

In summary, this study shows that irregularities along the circumference of lens capsules differ in quantity and appearance across different laser systems. Our results suggest that with current laser devices, differences in the irregularities seen along the capsulotomy margin are mainly attributable to variations in precision between different laser platforms, rather than to the amount of pulse energy applied. The classification of existing laser devices into high-energy lasers and low-energy lasers, thus, seems questionable because it assumes that pulse energy is the main determinant for an even capsulotomy and for rhexis stability. Rather than categorizing a laser device by pulse energy, we suggest individually evaluating anterior lens capsules to determine the effect a laser system has on the regularity of capsulotomy.

**WHAT WAS KNOWN**

- Capsulotomies in FLACS exhibit microscopic irregularities along their circumference.
- The amount of pulse energy applied during FLACS is one of the main known determinants of capsulotomy regularity and strength.

**WHAT THIS PAPER ADDS**

- The imprecise application of laser pulses, rather than the amount of pulse energy, is responsible for the main irregularities seen along the capsulotomy margin.
- Different cataract surgery laser platforms produce morphologically different capsulotomy irregularities, which may influence capsulotomy strength differently.
- Victus creates the smoothest capsulotomy margins. Catalys samples display a fringe of unconnected laser pulses along the capsulotomy, and FEMTO LDV Z8 samples typically exhibit many additional rows of laser pulses, an irregularity which could compromise capsular strength.
